# The CebE/MsiK Transporter is a Doorway to the Cello-oligosaccharide-mediated Induction of *Streptomyces scabies* Pathogenicity

**DOI:** 10.1038/srep27144

**Published:** 2016-06-02

**Authors:** Samuel Jourdan, Isolde Maria Francis, Min Jung Kim, Joren Jeico C. Salazar, Sören Planckaert, Jean-Marie Frère, André Matagne, Frédéric Kerff, Bart Devreese, Rosemary Loria, Sébastien Rigali

**Affiliations:** 1 Centre for Protein Engineering, Integrative Biological Sciences (InBioS) Research Unit, University of Liège, Institut de Chimie B6a, B-4000, Liège, Belgium; 2 Department of Biology, California State University Bakersfield, Bakersfield, CA 93311-1022, USA; 3 Department of Plant Pathology, University of Florida, Gainesville, FL 32611-0180, USA; 4 Laboratory for Protein Biochemistry and Biomolecular Engineering, Department of Biochemistry and Microbiology, Ghent University, B-9000, Ghent, Belgium

## Abstract

*Streptomyces scabies* is an economically important plant pathogen well-known for damaging root and tuber crops by causing scab lesions. Thaxtomin A is the main causative agent responsible for the pathogenicity of *S. scabies* and cello-oligosaccharides are environmental triggers that induce the production of this phytotoxin. How cello-oligosaccharides are sensed or transported in order to induce the virulent behavior of *S. scabies*? Here we report that the cellobiose and cellotriose binding protein CebE, and MsiK, the ATPase providing energy for carbohydrates transport, are the protagonists of the cello-oligosaccharide mediated induction of thaxtomin production in *S. scabies*. Our work provides the first example where the transport and not the sensing of major constituents of the plant host is the central mechanism associated with virulence of the pathogen. Our results allow to draw a complete pathway from signal transport to phytotoxin production where each step of the cascade is controlled by CebR, the cellulose utilization regulator. We propose the high affinity of CebE to cellotriose as possible adaptation of *S. scabies* to colonize expanding plant tissue. Our work further highlights how genes associated with primary metabolism in nonpathogenic *Streptomyces* species have been recruited as basic elements of virulence in plant pathogenic species.

The bacterial genus *Streptomyces* mostly consists of soil saprophytes able to produce a large arsenal of biologically active metabolites^1^ and catabolic enzymes making them well adapted to life in challenging soil environments^2^,^3^. Many *Streptomyces* species are able to either interact directly with plants or have an indirect positive effect on plant health and growth through mechanisms like biofertilization and biocontrol^4^,^5^,^6^. However, a selected group of *Streptomyces* species including *Streptomyces scabies, Streptomyces acidiscabies, Streptomyces turgidiscabies* and *Streptomyces ipomoeae* causes raised or pitted scab lesions on economically-important root and tuber crops like potato, radish, beet, peanut, and sweet potato^7^. These pathogenic species produce a family of non-ribosomally produced cyclic 2,5-diketopiperazines called thaxtomins. These phytotoxins primarily target the plant cell wall in dividing and expanding cell tissue through an alteration of expression of cell wall biosynthesis-related genes and depletion of cellulose synthase complexes from the plasma membrane^8^,^9^.

Thaxtomin A is the predominant form produced by *S. scabies, S. acidiscabies*, and *S. turgidiscabies*. Its production is under strict control involving several layers of regulation. At least five global regulators belonging to the *bld* gene family directing secondary metabolism and/or morphological differentiation of *Streptomyces* are involved in the regulation of toxin production^10^ in addition to the pathway-specific transcriptional activator, TxtR^11^. Moreover, we recently identified a key role for the cellulose utilization regulator, CebR, in unlocking the *S. scabies* pathogenicity^12^. CebR has been shown to bind upstream as well as within the thaxtomin biosynthetic cluster, and specific interaction between CebR and cellobiose or cellotriose was shown to trigger the release of the repressor from its binding sites hereby allowing transcription of the thaxtomin biosynthetic (*txtA* and *txtB*) and regulatory (*txtR*) genes^12^. These findings underline the significance of cellobiose and cellotriose as signal molecules for virulence besides serving as a nutrient^13^.

After having described how cellobiose and cellotriose unlock production of thaxtomin A^12^, we investigated and describe in this study how *S. scabies* links extracellular plant material sensing to the onset of its virulent behavior.

## Results

### *In silico* identification of the predicted cello-oligosaccharide transporter in *S. scabies*

**Figure 1:**
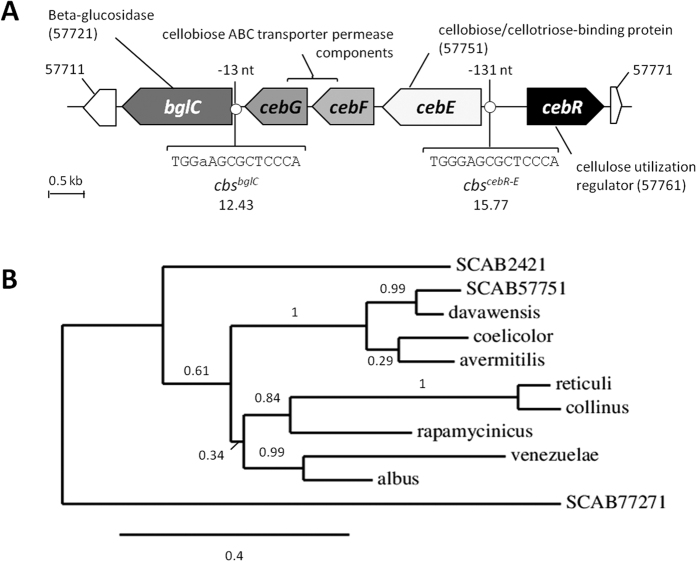
Organization of the putative cellobiose ABC transporter gene cluster and phylogeny analysis of CebE orthologues. (**A**) The *cebR*-*cebEFG*-*bglC* cluster in *S. scabies* 87–22 and positions of CebR-binding sites (*cbs*). Numbers associated with genes/ORFs are SCAB numbers from the annotated genome of *S. scabies* 87–22. The lower case letters indicate nucleotides that do not match with the *cbs* consensus sequence. (**B**) NJ tree with a series of orthologues of the CebE protein of *S. reticuli* (GI:5327251) including the three closest matches identified in the *S. scabies* chromosome (SCAB57751, SCAB2421, and SCAB77271). Other selected CebE proteins from *Streptomyces* species are: *albus* (GI:749175110), *avermitilis* (SAV5256, GI:29608916), *coelicolor* (SCO2795, GI:10303266), *collinus* (GI:529198022), *davawensis* (GI:505473507), *rapamycinicus* (GI:521359306), and *venezuelae* (SVEN_2583, GI:328882630). Percentage bootstrap values are shown at branch points. Bar, 0.4 substitutions per nucleotide position.

Earlier work in other *Streptomyces* model species showed that the CebEFG-MsiK ABC transporter is specifically involved in the uptake of cellobiose and cellotriose^14^,^15^,^16^. CebE is the solute-binding protein which binds cellobiose and cellotriose and therefore determines the specificity of the ABC transporter^17^, whereas CebF and CebG are the transmembrane proteins that line the pore across the bacterial cell membrane. ATP hydrolysis is mediated through the action of MsiK which is the multiple sugar import ATPase associated with many different ABC transporters in streptomycetes^16^,^18^,^19^,^20^. Orthologues of the genes/proteins involved in cello-oligosaccharide transport in *S. scabies* were identified by a BLAST search and by using the Absynte software^21^; both of which identified *scab57751, scab57741, scab57731*, and *scab50161* as the orthologues of *cebE, cebF, cebG*, and *msiK*, respectively. The genes encoding the transporter components (*cebEFG*) are organized in an operon (Fig. 1A), except for the gene that codes for the ATPase (*msiK*) which is located on another part of the chromosome (see Supplementary Figs S1 and S2). The *cebEFG* operon is divergently oriented from *cebR* encoding the cellulose utilization repressor^22^,^23^ and which also directly controls the expression of the thaxtomin biosynthetic (*txtA* and *txtB*) and regulatory genes (*txtR*)^12^. The gene coding for the predicted β-glucosidase (*bglC*) for further catabolism of the incoming cello-oligosaccharides into glucose is located 146 nt downstream of the tri-cistronic *cebEFG* operon. The synteny of the *cebR*-*cebEFG*-*bglC* cluster is conserved in most *Streptomyces* species (see Supplementary Fig. S1).

Earlier investigations identified two other putative ABC-type solute-binding proteins that could be involved in cello-oligosaccharide transport in *S. scabies*^24^, namely the products of genes *scab2421* and *scab77271*. However, phylogeny and synteny analyses suggest that SCAB57751 (CebE^*scab*^) is the orthologue of CebE of *S. reticuli* (CebE^*reti*^) which has been experimentally demonstrated to be the cellobiose and cellotriose-binding protein (Fig. 1B and see Supplementary Fig. S1). In addition, the *in silico* prediction of the CebR regulon identified the perfect palindromic TGGGAGCGCTCCCA CebR-binding site (*cbs*) at position −131 nt upstream of *scab57751* and another predicted *cbs* at position −14 nt upstream of *scab57721*, further supporting the hypothesis of the involvement of this gene in cellobiose utilization (Fig. 1A). Overall, the compilation of our *in silico* analyses strongly supports that *scab57751 (cebE*^*scab*^), *scab57741 (cebF*^*scab*^), *scab57731 (cebG*^*scab*^), and *scab50161 (msiK*^*scab*^) are the best candidate genes to encode the proteins of the cello-oligosaccharide-mediated induction of thaxtomin production in *S. scabies*.

### Cellobiose and cellotriose interact with CebE of *S. scabies* at a nanomolar range

To assess if CebE^*scab*^ can bind cellobiose and/or other cello-oligosaccharides, we used the intrinsic fluorescence of the aromatic residues of the protein to monitor conformational changes upon substrate binding. CebE^*scab*^ includes 10 tryptophan residues (in addition to 12 tyrosine and 13 phenylalanine residues) which exhibit a fluorescence signal at a wavelength between 300–400 nm when excited at 280 nm. Modeling of the 3D structure of the CebE protein with the YASARA software^25^ revealed conservation of the tryptophan residue predicted to participate in the binding of carbohydrates in ABC-type sugar-binding proteins, i.e. Trp303 within the binding site of the native protein (see Supplementary Fig. S3). As the environment of Trp303 is likely to be modified upon sugar binding, it strengthens the rationale of using intrinsic fluorescence as a methodology to assess CebE/cello-oligosaccharide interactions.

**Figure 2:**
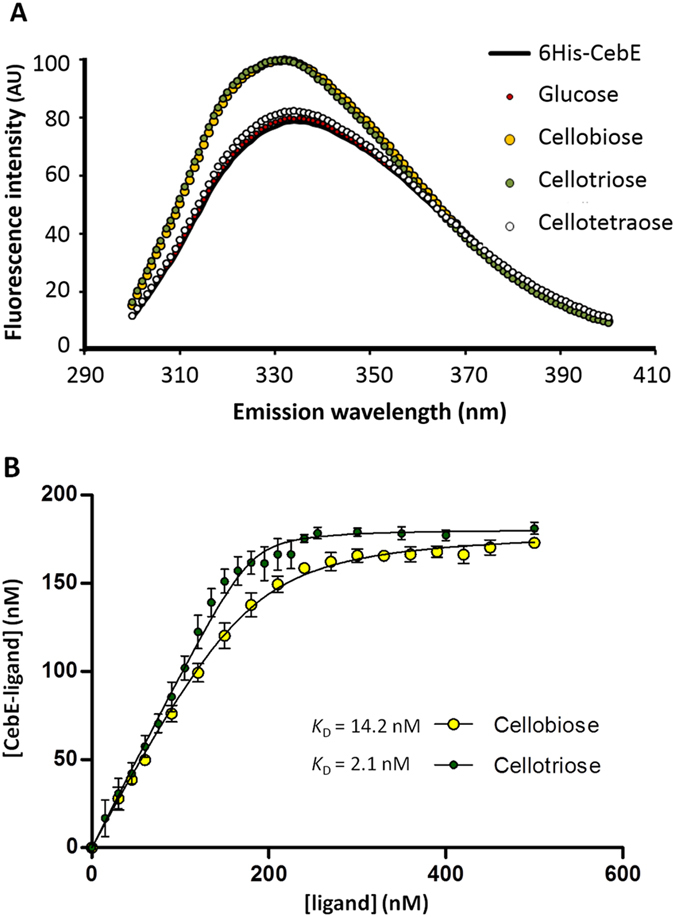
CebE of *S. scabies* interacts with cellobiose and cellotriose in the nanomolar range. (**A**) Fluorescence emission spectra of the pure 6His-CebE protein (180 nM) alone (line) or in presence of 30 μM glucose (red circles), cellobiose (yellow circles), cellotriose (green circles) or cellotetraose (white circles). (**B**) CebE-sugar dissociation curves as a function of the cellobiose or cellotriose concentration. The fluorescence emission intensity of 180 nM 6His-CebE was measured in presence of different concentrations of cellobiose or cellotriose (from 15 to 500 nM). Each dissociation experiment was repeated 6 times. See materials and methods for details.

The *cebE*^*scab*^ gene (*scab57751*) - without its first 44 codons encoding the peptide secretion signal and the membrane-anchoring lipopeptide - was cloned into pET-28a allowing for the production of an N-terminally 6 Histidine-tagged CebE protein (6His-CebE) in *Escherichia coli* and its subsequent purification through Ni-NTA affinity chromatography (see experimental procedures for details and Tables 1, 2 and Supplementary Fig. S4). We assessed the fluorescence emission spectra of 6His-CebE (100 nM) in the presence or absence of 30 μM glucose (Glc), cellobiose [(Glc)_2_], cellotriose [(Glc)_3_], cellotetraose [(Glc)_4_], cellopentaose [(Glc)_5_], cellohexaose [(Glc)_6_], as well as with 1 mM of other mono- and disaccharides unrelated to the cellulose utilization pathway (Fig. 2A and Supplementary Fig. S5). The addition of (Glc)_2_ and (Glc)_3_ resulted in an increase of fluorescence intensity, with a moderate ‘blue’-shift of the λ_max_ from 328 nm to 325 nm, indicating a modification of the environment of the tryptophan residues following interaction with cello-oligosaccharides (Fig. 2A). In contrast, the addition of glucose and cello-oligosaccharides (Glc)_4_, (Glc)_5_, and (Glc)_6_ neither significantly altered the fluorescence intensity between 300 and 400 nm nor caused a shift of the λ_max_ of 6His-CebE (Fig. 2A and Supplementary Fig. S5). The disaccharides maltose and trehalose also did not modify the fluorescence properties of 6His-CebE (not shown).

To determine if changes in intrinsic fluorescence intensity were due to a specific binding of (Glc)_2_ and (Glc)_3_, we calculated the dissociation constants (*K*_D_) by measuring the intrinsic fluorescence intensity of the protein in the presence of increasing concentrations of (Glc)_2_ and (Glc)_3_ (Fig. 2B). *F*_max_ could be estimated by addition of a large excess of (Glc)_2_ and (Glc)_3_ in the protein solution. Equations (1) and (2) (see Materials and Methods) were used to transform the raw fluorescence data and estimate the dissociation constants. The *K*_D_ values of cellobiose-CebE and of cellotriose-CebE complexes were determined as 14 (±2) nM and 2 (±0.5) nM, respectively (Fig. 2B). It is important to note that the *K*_D_ values of CebE^*scab*^ for (Glc)_2_ and (Glc)_3_, both in the nanomolar range, are 2 and about 3 orders of magnitude lower than the *K*_D_ values measured for the CebE protein of the cellulolytic species *S. reticuli* (~1.5 μM)^14^. The very high affinity of CebE to cellotriose could be a key adaptation of *S. scabies* allowing colonization of root and tuber crops (see Discussion).

### Effect of the inactivation of *cebE* and *msiK* on thaxtomin production and virulence of *S. scabies*

In order to know whether the cellobiose-mediated induction of thaxtomin A involves the (Glc)_2_ and (Glc)_3_-binding protein CebE, we deleted *scab57751* by gene replacement with an apramycin resistance cassette as described previously^12^. Similarly, the *scab50161* gene was deleted to investigate if the ATPase MsiK is involved in the uptake of (Glc)_2_ and (Glc)_3_. Oligonucleotides, constructs and protocols used for gene replacement are described in the Materials and methods section (Tables 1 and 2).

**Figure 3:**
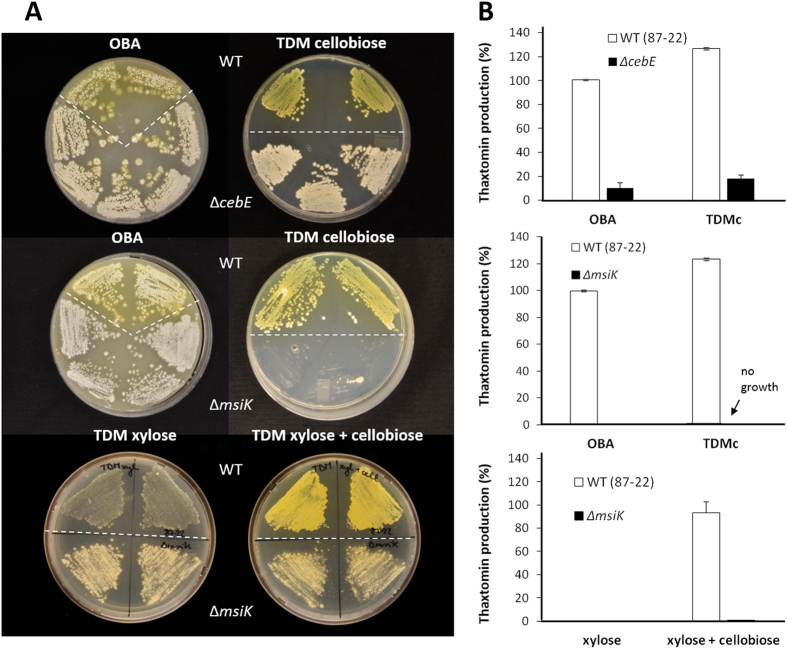
Effect of *cebE* and *msiK* deletion in *S. scabies* 87–22 on the production of thaxtomin. (**A**) Growth and thaxtomin production (yellow pigmentation) on plates. Xylose and cellobiose were both supplied at 1% final quantity (weight/volume percentage concentration). (**B**) HPLC analysis of thaxtomin extracted from plates. Deletion of *cebE* or *msiK* in *S. scabies* resulted in the loss of the cellobiose mediated induction of thaxtomin production.

The effect of *cebE* and *msiK* deletion on thaxtomin production was first assessed by visualization of the bright yellow color associated with this toxin. The *cebE* mutant was grown on OBA plates (complex medium which induces thaxtomin production) and on TDM (minimal medium) supplied with cellobiose as carbon source. As shown in Fig. 3A, this mutant lost the cellobiose-dependent induction of thaxtomin compared to the wild-type strain *S. scabies* 87–22. Surprisingly, the *cebE* mutant plated on solid TDM supplied with cellobiose as sole carbon source (TDMc) could still grow suggesting the existence of an additional uptake system for cellobiose in *S. scabies*, not involved in the thaxtomin production pathway. However almost no growth was detected when liquid TDMc was inoculated with this mutant (see Supplementary Fig. S6). In contrast, the *msiK* mutant was unable to grow on or in TDMc (Fig. 3A). This implies that if *S. scabies* does possess multiple cellobiose transporters, they all require MsiK to drive the import. To verify that deletion of the *msiK* gene would lead to loss of the cellobiose-dependent induction of thaxtomin, this mutant was further plated on TDM supplied with xylose (with and without cellobiose supply, abbreviated TDMx and TDMxc, respectively), a carbohydrate reported to be a carbon source that does not fully require MsiK-dependent uptake^18^. Indeed, as shown in Fig. 3A, the *msiK* mutant was able to grow with xylose as sole carbon source, but was unable to produce thaxtomin in response to the presence of cellobiose.

Extraction of the toxin from different media and semi-quantitative analysis by high-performance liquid chromatography (HPLC) revealed that only 10 and 20% of thaxtomin production remained for the *cebE* mutant grown on OBA, and TDMc, respectively, compared to the parental strain 87–22 (Fig. 3B). No thaxtomin production could be detected for the *msiK* mutant on OBA, TDMx, and only 2% of thaxtomin on TDMxc compared to the wild type strain *S. scabies* 87–22 (Fig. 3B).

**Figure 4:**
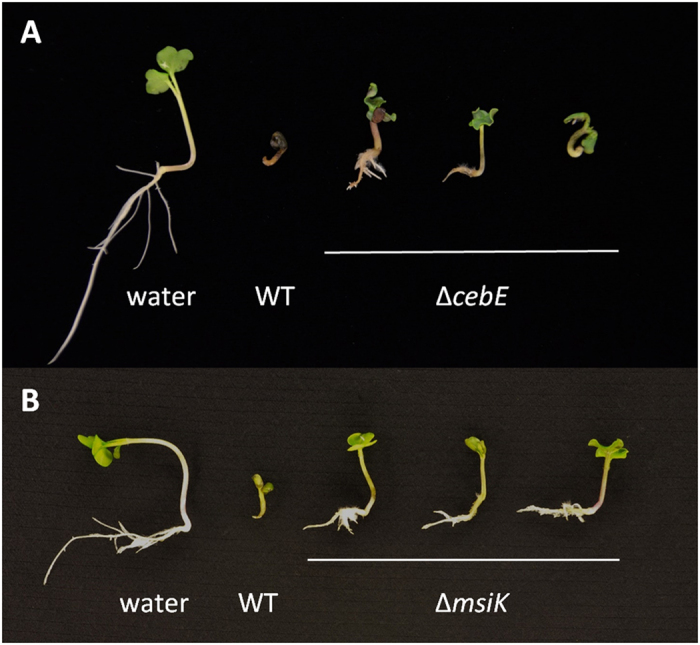
Effect of *cebE* and *msiK* deletion on the virulence of *S. scabies*. Phenotypes of representative radish seedlings are shown after 7 days of growth treated with water, the wild type strains 87–22, and the *cebE* deletion mutant (**A**) or the *msiK* deletion mutant (**B**).

We further evaluated the effect of *cebE* and *msiK* deletion on the virulence of *S. scabies* on radish seedlings. *S. scabies* typically causes severe root and shoot stunting, tissue swelling as well as necrosis, especially at the root tip (Fig. 4). These symptoms are typically attributed to thaxtomin production^26^. Both mutants showed an attenuated virulence phenotype when inoculated onto radish seedlings compared to the wild type strain (Fig. 4A,B).

Overall, these results (Figs 3 and 4) demonstrate that transport and not sensing of cellobiose is required for the induction of thaxtomin biosynthesis and thus the virulent behavior of *S. scabies* 87–22.

### The *cebEFG* operon is repressed by CebR and induced by cellobiose

**Figure 5:**
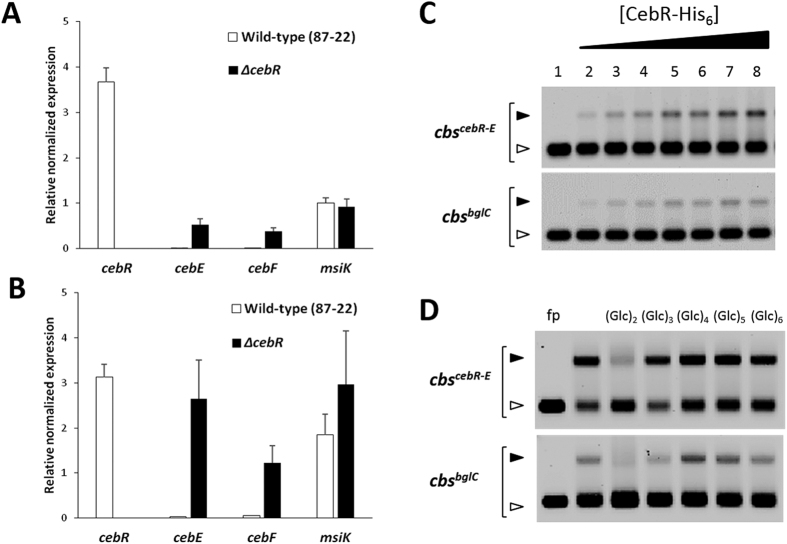
CebR directly represses transcription of genes encoding components of the cellobiose/cellotriose-specific ABC transporter. (**A**,**B**) Effect of *cebR* deletion in *S. scabies* on the transcription levels of *cebE, cebF*, and *msiK* when grown on ISP-4 (**A**) and OBA (**B**). qPCR analysis of gene expression levels in *S. scabies* 87–22 and in the Δ*cebR* strain. Data were normalized using the *gyrA* and *murX* genes as internal controls. Mean normalized expression levels (±standard deviations) from three biological repeats analyzed in triplicate are shown. *Denotes significant quantitative overexpression (p > 0.05) in the *S. scabies cebR* null mutant compared to the wild-type strain 87–22. (**C**) CebR binds to the DNA motifs identified in the intergenic region between *cebR* and *cebE (cbs*^*cebR-E*^) and upstream of *bglC (cbs*^*bglC*^) (see Fig. 1A for sequences and localization of *cbs*). EMSAs with increasing concentrations of pure CebR-His6, i.e., 0 30, 80, 160, 240, 320, 400, and 480 nM, respectively (numbers 1 to 8). (**D**) EMSAs demonstrating that cellobiose and cellotriose are able to inhibit the DNA-binding ability of CebR. Numbers 1 and 2 refer to EMSAs with free probes (6 nM) and with probes incubated with CebR-His6, respectively. Numbers 3 to 7 refer to EMSAs with CebR-His6 preincubated with oligosaccharides, i.e., cellobiose (lane 3), cellotriose (lane 4), cellotetraose (lane 5), cellopentaose (lane 6), or cellohexaose (lane 7).

To further support the involvement of SCAB57751 (CebE^*scab*^) in cello-oligosaccharide utilization we investigated how the expression of the *cebEFG* operon was controlled by the cellulose utilization repressor CebR. Quantitative real-time reverse transcription-PCR (qPCR) was performed on RNA extracted from the *S. scabies* wild-type (strain 87–22) and its *cebR* mutant grown on ISP-4 and OBA. On both media we observed that the deletion of *cebR* resulted in overexpression of *cebE* and *cebF*, whereas the expression of *msiK* was insignificantly altered compared to the wild-type levels (Fig. 5A,B). The transcriptional control of CebR on *cebEFG* expression is mediated through a direct interaction with the *cis*-acting elements located at positions −131 and −14 nt upstream of *cebE* and *bglC*, respectively (Fig. 5C,D).

**Figure 6:**
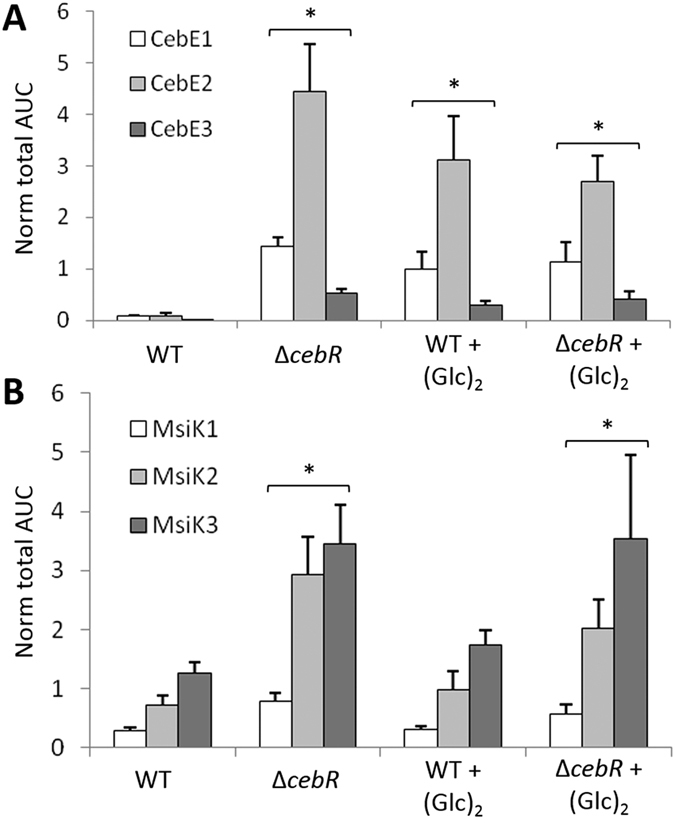
Relative abundancy of CebE (**A**) and MsiK (**B**) peptides in response to the deletion of *cebR* and/or cellobiose supply, determined by targeted proteomics (LC-MRM). Target peptides for CebE: ESDYLPWK (CebE1), SAFDLTAK (CebE2), and SGNWGGSFLSVPK (CebE3). Target peptides for MsiK: ILDLTEYLDR (MsiK1), TQIASLQR (MsiK2), and FGNSVVPVNR (MsiK3). *Denotes significant quantitative peptide overproduction (P < 0.05) compared to the wild-type strain grown in ISP-4 without cellobiose supply. Statistical significance was assigned by performing 2-sided Student’s t-tests and assuming groups of equal variances (See Supplementary Fig. S7).

The effect of the deletion of *cebR* as well as the presence of cellobiose on the production of the CebE-MsiK transporter was further assessed with a label-free, targeted liquid chromatography-multiple reaction monitoring (LC-MRM) approach. Three proteotypic peptides for CebE, and MsiK were quantitatively analyzed by MRM: ESDYLPWK, SAFDLTAK and SGNWGGSFLSVPK for CebE, and ILDLTEYLDR, TQIASLQR and FGNSVVPVNR for MsiK. Peptides from CebE were overrepresented in extracts of the *cebR* deletion mutant or when the wild-type strain 87–22 was cultivated in the presence of cellobiose (Fig. 6A). Surprisingly, while we could not detect overexpression of *msiK* transcripts trough the qPCR approach (Fig. 5A), peptides from MsiK were 3-fold overrepresented in the ∆*cebR* background whereas their quantity was not increased upon cellobiose supply (Fig. 6B). The deletion of *cebR* had a greater effect on the overproduction of CebE and MsiK than the cultivation of *S. scabies* 87–22 on ISP-4 medium supplied with cellobiose. In agreement with their role in the virulence of *S. scabies*, the genes for the cellobiose and cellotriose *cebEFG* transporter are induced by the incoming carbohydrates and repressed by CebR.

## Discussion

**Figure 7:**
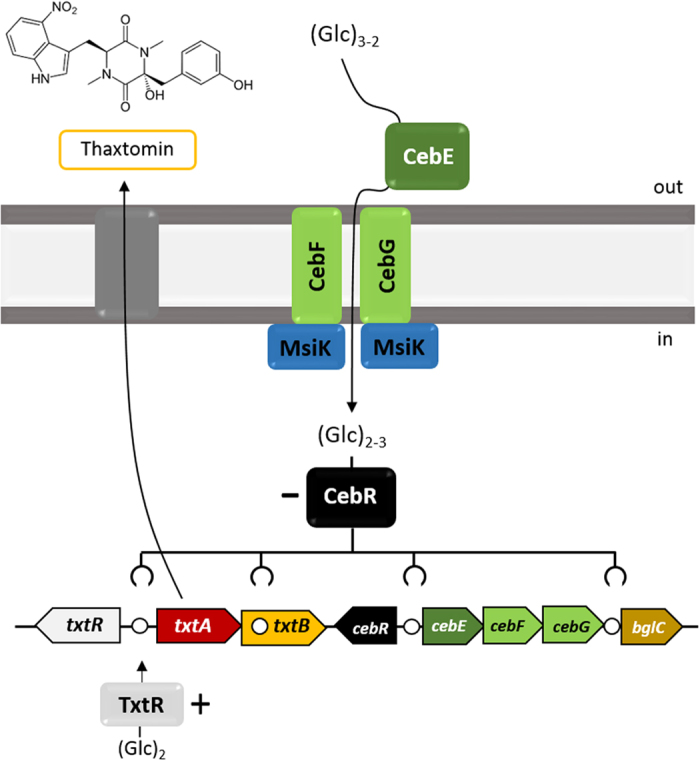
Model of the cellobiose/cellotriose-mediated induction of thaxtomin production in *S. scabies*. Cellobiose (Glc)_2_ and Cellotriose (Glc)_3_ are transported by the CebEFG-MsiK ABC transporter. In the cytoplasm, (Glc)_2_ and, to a lesser extend (Glc)_3_, binds to CebR and inhibits its DNA-binding ability which further increases transcription of *cebE, cebF*, and *cebG*, as well as *txtR*, the specific activator of the thaxtomin A biosynthetic genes *txtA* and *txtB*. Circles (○) indicate the CebR-binding sites.

In this work we show that the cello-oligosaccharide-induced pathogenicity of *S. scabies* 87–22 is mediated, at least in great part, through the CebEFG-MsiK ABC transporter system. This allows to propose a first signaling pathway from cello-oligosaccharide transport to the production of thaxtomin A in *S. scabies*, with each step of the cascade controlled by the cellulose utilization transcriptional repressor CebR (Fig. 7).

Only very few other (plant) pathogens have been shown to also use carbohydrates from the host as signals to boost their virulence behavior. The best studied phytopathogenic bacterium *Agrobacterium tumefaciens* recognizes its host environment by binding to diverse aldose monosaccharides as well as sugar acid derivatives through the periplasmic protein ChvE^27^,^28^,^29^. ChvE, like CebE, is a carbohydrate-binding protein associated with an ABC transporter but the molecular mechanisms involved in triggering virulence highly differ from the one highlighted in *S. scabies*. The ChvE-sugar complex binds to the periplasmic domain of the two-component system sensor VirA making it more sensitive to plant phenolic compounds which ultimately leads to virulence (*vir*) genes expression^27^,^28^,^29^,^30^,^31^. In the *A. tumefaciens*-plant interaction, the carbohydrates emanating from the host thus participate in a three-component sensing system instead of being directly imported into the cytoplasm to target the master regulator of the process as highlighted in *S. scabies*.

Importantly, CebE of *S. scabies* displays binding affinities for cellobiose and cellotriose which are, respectively, about 2 and 3 orders of magnitude greater than the affinity measured for CebE of the highly cellulolytic species *S. reticuli (K*_D_ ~ 1.5 μM)^14^. The fact that *S. scabies* displays no or very poor growth on minimal media with carboxymethyl cellulose, fibrous cellulose or microcrystalline cellulose as unique carbon source^13^, whereas it responds to such low concentrations of cellobiose and cellotriose has most likely an important biological meaning. This suggests that *S. scabies* could possibly behave as a ‘cheating’ organism in soils where intense degradation of abundant plant biomass occurs, and be one of the first beneficiaries of the cello-oligosaccharides generated by efficient lignocellulolytic microorganisms. However, transport and utilization of cellobiose and cellotriose released from cellulolytic microorganisms would also trigger production of the phytotoxin thaxtomin under conditions where it is useless as the large majority of the plant material is already dead and decomposing. This is probably why additional levels of control of thaxtomin production are required. One of these is the post-transcriptional control by the *bldA* gene which encodes for the leucyl tRNA of the rare UUA codon which is notably found within the sequence coding for the thaxtomin production transcriptional activator TxtR^11^ and the BldH regulator also required for thaxtomin production and plant virulence^10^. Since this leucyl tRNA is only abundant at late exponential phase in streptomycetes^32^, this post-transcriptional level of control would guarantee the delay of the production of thaxtomin until *S. scabies* has reached a certain density.

Alternatively, this high responsiveness to very low concentrations of cellobiose and cellotriose could suggest a more appropriate fitness for environments where the abundance of these molecules is expected to be more marginal such as in the diffusion zone of root exudates. Interestingly, Johnson *et al*. detected cellotriose (but not cellobiose) released from rapidly growing radish seedling and from growing tobacco NT1 cell suspensions^13^ and we show here that the affinity of CebE of *S. scabies* for cellotriose is greater of about one order of magnitude compared to cellobiose. The higher sensitivity to cellotriose could be an adaptation to the environment where it is appropriate for *S. scabies* to sense and respond to a specific composition and concentration of cello-oligosaccharides. Indeed, cellotriose could be the plant signalling molecule detected by *S. scabies* indicating the presence of a growing host in its surrounding. Sensing cellotriose (in a cellobiose free environment) could mean sensing a signal from an expanding plant tissue that also constitutes the site of invasion of *S. scabies* as well as the site of action of thaxtomin. Johnson *et al*. also showed that the phytotoxin increases the release of cello-oligosaccharides by expanding plant tissues. So, a small amount of cellotriose in the soil would be sufficient to initiate the production of low amounts of thaxtomin and the latter would in turn target the plant cellulose synthase resulting in a more abundant release of cellotriose that, once imported by the CebEFG-MsiK transporter, would increase thaxtomin biosynthesis. Instead of active cellulases, thaxtomin itself would generate its own trigger, cellotriose, as postulated earlier^13^.

Further structural comparative studies are required to understand why CebE of *S. scabies* 87–22 displays such a high affinity for cellotriose and, importantly, if this is a feature conserved in CebE proteins of other plant pathogens such as *S. acidiscabies*, and *S. turgidiscabies* that could also have elaborated the onset of their virulent behaviour upon cello-oligosaccharides uptake. Finally, at this stage, we cannot exclude that other proteins may also participate in the transport and/or the sensing of cello-oligosaccharides. Indeed, proteomic investigations in *S. scabies* EF-35 suggest that suberin may also participate in the induction of the cellulose/cello-oligosaccharides utilization regulon^24^,^33^,^34^,^35^.

## Materials and Methods

### Bacterial strains and culture conditions

All strains and plasmids used in this study are described in Table 1. *Escherichia coli* strains were cultured in Luria-Bertani (LB) medium at 37 °C. *Streptomyces* strains were routinely grown at 28 °C in tryptic soy broth (TSB; BD Biosciences) or on International *Streptomyces* Project medium 4 (ISP-4, BD Biosciences). When required, the medium was supplemented with the antibiotics apramycin (100 μg/ml), kanamycin (50 μg/ml), chloramphenicol (25 μg/ml), thiostrepton (25 μg/ml), and/or nalidixic acid (50 μg/ml). Cellobiose and cello-oligosaccharides were purchased from Megazyme (Ireland).

### Production and purification of histidine-tagged recombinant CebE protein

The open reading frame encoding SCAB57751 (CebE) without its first 132 nucleotides was amplified by PCR using primers *scab_*57751_+132_NdeI and scab_57751_+1365_HindIII (see Table 2). The corresponding PCR product was subsequently cloned into the pJET1.2/blunt cloning vector, yielding pSAJ015. After DNA sequencing to verify the correct amplification of *scab57751*, an NdeI-HindIII DNA fragment was excised from pSAJ015 and cloned into pET-28a digested with the same restriction enzymes leading to pSAJ016. *E. coli* Rosetta^TM^ (DE3) cells carrying pSAJ016 were grown at 37 °C in 250 ml LB medium containing 100 μg/ml of kanamycin until the culture reached an absorbance at 600 nm (A_600_) of 0.6. Production of 6His-tagged CebE (6His-CebE) was induced overnight (~20 h) at 16 °C by addition of 1 mM isopropyl-β-D-thiogalactopyranoside (IPTG). Cells were collected by centrifugation and ruptured by sonication in lysis buffer (100 mM Tris-HCl buffer; pH 7.5; NaCl 250 mM; 20 mM imidazole 20 mM supplemented with the EDTA-free cOmplete protease inhibitor cocktail (Roche). Soluble proteins were loaded onto a pre-equilibrated Ni^2+^-nitrilotriacetic acid (NTA)-agarose column (5-ml bed volume), and 6His-CebE eluted with 90 to 200 mM imidazole. Fractions containing the pure protein were pooled (see Supplementary Fig. S4) and dialyzed overnight in 30 mM Tris-HCl buffer; pH 7.5; NaCl 150 mM.

### Tryptophan Intrinsic fluorescence assays

Pure 6His-CebE was dissolved in a 12 mM Tris-HCl buffer pH 7.5; 40 mM NaCl to a final concentration of 180 nM and analyzed at room temperature by a Cary Eclipse spectrofluorimeter (Varian Ltd). Measurements were performed with 1 ml of sample mix in a 10 mm path quartz cuvette with an excitation wavelength of 280 nm. Each emission fluorescence spectrum is the mean of 10 scans acquisitions between 300 and 400 nm at a speed of 600 nm/minute. Excitation and emission slit lengths were 5 and 10 nm respectively. The potential difference of the photomultiplicator was fixed at 750 V. To assess the binding ability of different sugars to 6His-CebE, we first compared the fluorescence emission spectra of the pure 6His-CebE (180 nM) with or without 30 μM glucose, maltose, trehalose, cellobiose, cellotriose, cellotetraose, cellopentaose, or cellohexaose.

Determining of dissociation constants (*K*_D_) from fluorescence data:

Samples used to determine the dissociation constants (*K*_D_) were prepared and analyzed as described above with 25 different concentrations (ranging from 15 nM to 500 nM) of cellobiose or cellotriose. Fluorescence assays were performed with six different preparations of pure 6His-CebE protein.

The proportion of 6His-CebE bound to cello-oligosaccharide was calculated using the following equation:


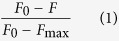


where 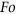 is the fluorescence intensity of the free 6His-CebE protein, 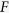 is the fluorescence of 6His-CebE in the presence of a given cello-oligosaccharide concentration, and 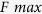 is the fluorescence of 6His-CebE in the presence of excess sugar (saturating conditions).

These data were introduced in the GraphPad Prism version 5.0 (GraphPad Software, San Diego California USA) and the following nonlinear curve fitting function was used to find the dissociation constant *K*_D_ using equation (2):


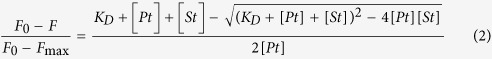


In which 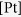 is the total 6His-CebE concentration and 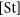 is the total sugar (cello-oligosaccharide) concentration.

### Electromobility gel shift assays (EMSAs)

Interaction studies between pure His-tagged CebR and DNA probes with the predicted CebR-binding sites (*cbs*) were performed by EMSAs as described previously^12^, using 15 pmol of Cy5-labelled probes and 150 pmol of CebR-6His. EMSA reactions were loaded into a 1% (w/v) agarose gel and bound and unbound probes were separated by gel electrophoresis at room temperature. Fluorescent Cy5-labbelled DNA was visualized using a Typhoon Trio+ Variable Mode Imager (GE Healthcare, laser excitation at 633 nm and emission filter at 670 nm (BP30)). The sequences of oligonucleotides used to generate the *cbs* probes are listed in Table 2.

### Construction of *cebE* and *msiK* null mutants

Deletion mutants in *S. scabies* 87–22 were created using the REDIRECT^©^ PCR targeting methodology^36^ that replaces the selected gene by an antibiotic deletion cassette as previously described for *cebR*^12^. These cassettes consisting of an *oriT* and an antibiotic resistance gene (*aac*(*3*)*IV* for apramycin resistance, Table 1) and flanked by FRT sites (FLP-recombinase recognition targets), were generated by PCR using primers with gene-specific homology extensions (Table 2) and pIJ773 (Table 1) as template. The gel-purified deletion cassettes were electroporated into the *E. coli* BW25113 strain harboring the arabinose-inducible λ RED expression plasmid, pIJ790, and a cosmid containing the gene of interest. Transformants were recovered on apramycin selective medium, and correct gene replacement in the cosmid was confirmed by PCR and sequencing. The resulting mutated cosmid was then transferred to *S. scabies* 87–22 via intergeneric conjugation after passage through the *E. coli* ET12567 strain harboring pUZ8002 (Table 1). Exconjugants were selected for resistance to apramycin, and sensitivity to kanamycin. Genomic DNA was extracted from *Streptomyces* cultures grown in TSB medium using the MasterPure™ Gram Positive DNA Purification Kit (Epicentre Biotechnologies) according to the manufacturer’s instructions and verification of the mutant isolates was performed by PCR.

### Quantitative Real-Time PCR

RNA was prepared from 72-h old mycelia grown on ISP-4 and TDMc at 28 °C using the RNeasy Mini Kit (Qiagen) according to the manufacturer’s instructions. PCRs on the purified RNA were performed to verify the absence of genomic DNA (data not shown). cDNA synthesis was performed starting from 1 μg of DNAse-treated (Turbo DNA-free Kit, Ambion) RNA using the iScript^TM^ cDNA Synthesis Kit (BioRad). Quantitative Real-Time PCR (qPCR) was carried out in a total volume of 10 μl containing 4 μl of SsoAdvanced^TM^ Sybr^®^ Green Supermix (BioRad), 4 μl of 1/10 diluted cDNA and 0.5 pmol of each gene-specific primer (Table 2), and subjected to the following PCR protocol: 3 min at 95 °C, 40 cycles of 30 s at 95 °C followed by 45 s at 60 °C. A melting curve analysis (samples were heated from 60 °C to 95 °C) was performed after each qPCR run to verify the specific amplification of each product. The *murX* and *gyrA* genes^11^ were used to normalize for the amount of RNA in the samples. Each measurement was performed in triplicate with three biological replicates per strain or condition.

### Targeted proteomic analysis

*S. scabies* 87–22 and its *cebR* null mutant were grown on ISP-4 plates with or without a 0.7% cellobiose supply. The mycelium was collected after 48 hours of incubation at 28 °C, suspended in 50 mM NH_4_HCO_3_ buffer (pH 7.5) and crude intracellular extracts were obtained after sonication of the mycelium as described previously^37^ Sample preparation for Liquid Chromatography-Multiple Reaction Monitoring (LC-MRM) analysis, LC-MRM analysis, and LC-MS^E^ analysis were performed as previously described^38^ and fully detailed in S1 File.

### Thaxtomin extraction and quantification

Mycelial suspensions of *S. scabies* strains (wild-type and different isolates of each mutant) were prepared from 48–72 h-old TSB-grown cultures by pelleting the mycelia, washing twice with sterile water, and resuspending in sterile water to an A_600_ of 1.0. Samples of 50 μl were plated out on small Petri dishes (5-cm diameter) containing 12.5 ml OBA or ISP-4 medium.

After incubation for 7 days at 28 °C, the medium was chopped into small cubes and soaked in 8 ml of methanol for 10 min. The supernatant was filtered through a 0.2-μm polytetrafluoroethylene (PTFE) filter and analyzed via HPLC on a Zorbax RX-C18 column (5 μm, 4.6 × 250 mm, Agilent Technologies) with a 1 ml/min flow rate of an isocratic mobile phase of 40:60 acetonitrile:water. Thaxtomin A was detected by measuring the absorbance at 380 nm. All experiments were repeated using different biological replicates of the *Streptomyces* strains, with three technical replicates per strain.

### Plant virulence assays

To assess the virulence phenotype of the *S. scabies cebE* and *msiK* mutants, an *in vitro* radish seedling assay was performed. Seeds of the “White Beauty” variety (Burpee) were surface sterilized for 5 min in 70% ethanol followed by a 10 min incubation in 15% (vol/vol) bleach. The seeds were allowed to germinate for about 30 h at 21 ± 2 °C in the dark in a Petri dish containing a moistened filter paper. Germinated radish seeds placed into six agar wells (13 mm in diameter) formed in an deep 1.5% agar-water plate were inoculated each with a 200 μl mycelial sample, prepared as described above, or sterile water as the control. The plates were incubated at 21 ± 2 °C under a 16-h photoperiod for 6 days. The assays were performed three times.

### Bioinformatics

A search for orthologues of CebE from *S. reticuli* (GI:5327251) in selected *Streptomyces* species and synteny analysis were performed using the Archaeal and Bacterial Synteny Explorer software (Absynthe) using the “best genomic match” search parameter at a 30% minimal score threshold (http://archaea.u-psud.fr/absynte/)^21^. Selected CebE homologues were used as a training set to generate the phylogenetic tree via the Phylogeny.fr platform^39^ using the “One click” mode which provides a ready-to-use pipeline including the following programs programs: MUSCLE for multiple alignment, Gblocks for automatic alignment curation, PhyML for tree building, and TreeDyn for tree drawing. CebE homologues used for the analysis are listed in the legend of Fig. 1B. The new CebR position weight matrix (see Supplementary Fig. S4) was created using DNA motifs known to be bound by CebR in *S. griseus*^22^ and *S. scabies*^12^ with the PREDetector software^40^ and the prediction of the CebR regulon was performed according to the philosophy described previously^41^. GraphPad Prism 5 software was used to determine binding parameters of CebE by fitting data obtained from the tryptophan intrinsic fluorescence analysis. Modeling of the 3D structure of the CebE protein was performed with the YASARA software^25^ using as template the crystal structure of the ABC transporter Solute Binding Protein from Thermotoga Lettingae TMO (Tlet_1705, TARGET EFI-510544) bound with alpha-D-Tagatose (5CI5). Because of the low sequence identity with the model (21.8%), the overall quality Z-score is only ranked as satisfactory (−1.929). The position of Trp303 in the binding pocket of CebE is clearly established as this residue is strictly conserved and well aligned in all structures used for molecular modelling. The figure displaying this structure was obtained using the program Pymol (The PyMOL Molecular Graphics System, Version 1.7.4.3 Enhanced for Mac OS X, Schrödinger, LLC.).

## Additional Information

**How to cite this article**: Jourdan, S. *et al*. The CebE/MsiK Transporter is a Doorway to the Cello-oligosaccharide-mediated Induction of *Streptomyces scabies* Pathogenicity. *Sci. Rep.* **6**, 27144; doi: 10.1038/srep27144 (2016).

## Supplementary Material

Supplementary Information

## Figures and Tables

**Table 1:** Bacterial strains, plasmids, and cosmids used in this study.

Plasmids, cosmids and strains	Description^†^	Source or reference
Plasmids or cosmids		
pJET1.2/blunt	*E. coli* plasmid used for high-efficiency cloning of PCR products (Amp^R^)	Thermo Scientific
pET28a	Expression vector used to produce N-terminal His-tagged protein in *E. coli* (Kan^R^)	Novagen
pSAJ002	pET22b derivative containing the *scab57761 (cebR*) coding sequence inserted into *Nde*I and *EcoR*I restriction sites (Amp^R^)	12
pSAJ015	pJET1.2 derivative containing the *scab57751* coding sequence without the first 132 nt (Amp^R^)	This study
pSAJ016	pET28a derivative containing the *scab57751 (cebE*) coding sequence without the first 132 nt inserted into *Nde*I and *Hind*III restriction sites (Kan^R^)	This study
pIJ790	λ Red plasmid (t^S^, Cml^R^)	36
pUZ8002	Supplies transfer functions for mobilization of *oriT*-containing vectors from *E. coli* to *Streptomyces* (Kan^R^)	42
pIJ773	Template for the REDIRECT^©^ PCR targeting system, contains the [*aac*(*3*)*IV*+*oriT*] disruption cassette (Amp^R^, Apr^R^)	36
SuperCos1	Cosmid cloning vector (Amp^R^, Kan^R^)	Stratagene
Cosmid 833	SuperCos1 derivative containing the *S. scabies* 87–22 cellobiose utilization regulator CebR locus (Kan^R^, Amp^R^)	This study
*E. coli* strains		
DH5α	General cloning host	Gibco-BRL
Rosetta (DE3)	*E. coli* strain used to express a protein from pET-vectors (Cml^R^)	Novagen
BW25113	Host for the REDIRECT^©^ PCR targeting system	36
ET12567	*dam*^*−*^*, dcm*^*−*^, *hsdS*^*−*^; non-methylating host for transfer of DNA into *Streptomyces* spp. (Cml^R^, Tet^R^)	36
*Streptomyces* strains		
87–22	*S. scabies* wild type strain	43
∆*scab57761* (∆*cebR*)	87–22 derivative with a deletion of the *scab57761* gene (Apr^R^)	12
∆*scab57751* (∆*cebE*)	87–22 derivative with a deletion of the *scab57751* gene (Apr^R^)	This study
∆*scab50161* (∆*msiK*)	87–22 derivative with a deletion of the *scab50161* gene (Apr^R^)	This study

^†^Cml^R^, chloramphenicol resistance; Kan^R^, kanamycin resistance; Amp^R^, ampicillin resistance Apr^R^, apramycin resistance; Tet^R^, tetracylcin resistance; t^s^, temperature sensitive.

**Table 2:** Primers used in this study.

Primers	Sequence (5′ → 3′)*	Application
Heterologous expression		
* scab_*57751 + 132 NdeI	*CATATG*GACGACGGCAAGGACGAGG	PCR for cloning *scab57751* in pET28a
* scab*_57751 + 1365 HindIII	*AAGCTT*TCACTTGTCCAGTGCGTTGTCG	
EMSA		
* scab*_57751c-140 Cy5	CCAGGTACTGTGGGAGCGCTCCCACGAGTGATGT	*cbs*^*cebE*^ probe
* scab*_57761 − 506	ACATCACTCGTGGGAGCGCTCCCACAGTACCTGG	
* scab*_57721+10	GGTTCAGGCATGGAAGCGCTCCCATTGGTGGTCG	*cbs*^*bglC*^ probe
* scab*_57721-24_Cy5	CGACCACCAATGGGAGCGCTTCCATGCCTGAACC	
Gene inactivation		
sc052	CGGGGGGTCGGCACGTCAGGGCATCAGGAGGACGCAATGATTCCGGGGATCCGTCGACC	*scab57751 (cebE*) Redirect deletion cassette
sc053	GGGGCGCGGCGGTGCGGGCCTTGTGGGCATACCGGTCATGTAGGCTGGAGCTGCTTC	
sc056	CTTACACATCCCGCACGTTCCC	PCR verification of ∆*scab57751 (cebE*)
sc057	TGTCGGGCCTTGTGGGCATA	
imf171	CGGCACGTTCCTGCCGGTGAAGGGGGCCTACGACCCATGATTCCGGGGATCCGTCGACC	*scab50161 (msiK*) Redirect deletion cassette
imf172	GGTTTCGCGCACGGTCACGGACGGGCCGTGCCCGGATCATGTAGGCTGGAGCTGCTTC	
imf177	TACCTGGAACTCGCCTGGTC	PCR verification of ∆*scab50161 (msiK*)
imf178	CATCCTGTTTGTCGGGGTAG	
Gene expression analysis		
imf200	CTGGGTTACGTCCCGAACAC	*scab57761 (cebR*)
imf201	CCTTGAGGATGTCGGAGAAG	
imf190	CCTCGGGCAAGGTCATCTAC	*scab57751 (cebE*)
imf191	GCTGGAACTGCGTCTGGTTC	
imf341	TCCAGGTGATCCCGCTCTAC	*scab57741 (cebF*)
imf342	CGCATGAAGAACACACCGAA	
imf257	AAGATCCTCGACCTCACCGA	*scab50161 (msiK*)
imf258	GTCCATGAGGAACACCTGGG	
DRB21	GTCTGGCAGTTCCAGGAGTC	*scab67971 (murX*)
DRB22	AGGTGTTCCACCACAGGAAG	
DRB23	GGACATCCAGACGCAGTACA	*scab45751 (gyrA*)
DRB24	CTCGGTGTTGAGCTTCTCCT	

^*^Engineered restriction sites are indicated in italic, CebR-binding sites are in bold, and non-homologous extensions are underlined.
