# Supplementary material for: The CebE/MsiK Transporter is a Doorway to the Cello-oligosaccharide-mediated Induction of Streptomyces scabies Pathogenicity
Source: Sci Rep. 2016 Jun 2;6:27144. doi: 10.1038/srep27144 (PMC4890002; doi:10.1038/srep27144)
Supplement: Supplementary Information [file srep27144-s1.pdf]

The CebE/MsiK Transporter is a Doorway to the Cello-oligosaccharide-mediated Induction of *Streptomyces scabies* Pathogenicity

Samuel Jourdan, Isolde Maria Francis, Min Jung Kim, Joren Jeico C. Salazar, Sören Planckaert, Jean-Marie Frère, André Matagne, Frédéric Kerff, Bart Devreese, Rosemary Loria, and Sébastien Rigali

Supplementary Figures

Supplementary Figure S1.

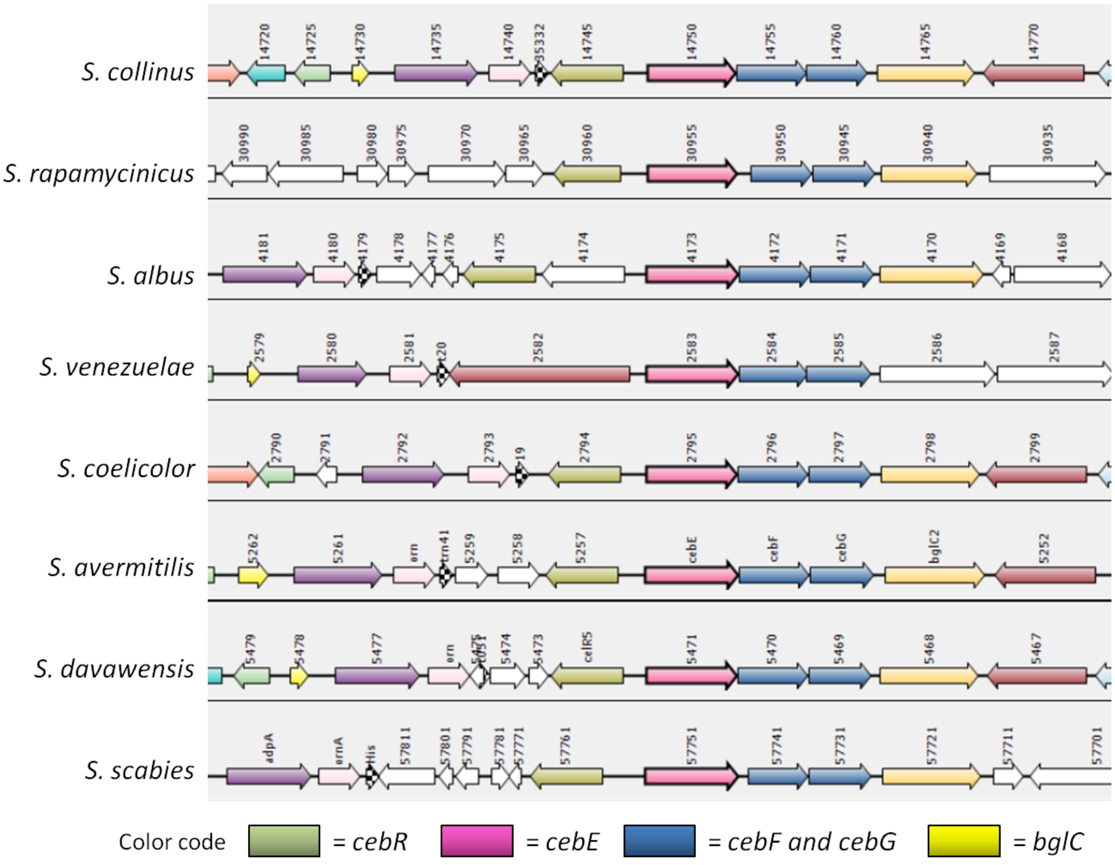

Synteny of the *cebR-cebEFG-bglC* cluster in a series of *Streptomyces* species. The amino acid sequence of CebE of *S. reticuli* was used as query. The Absynte software identified SCAB_57751 as closest match of CebE.

Supplementary Figure S2.

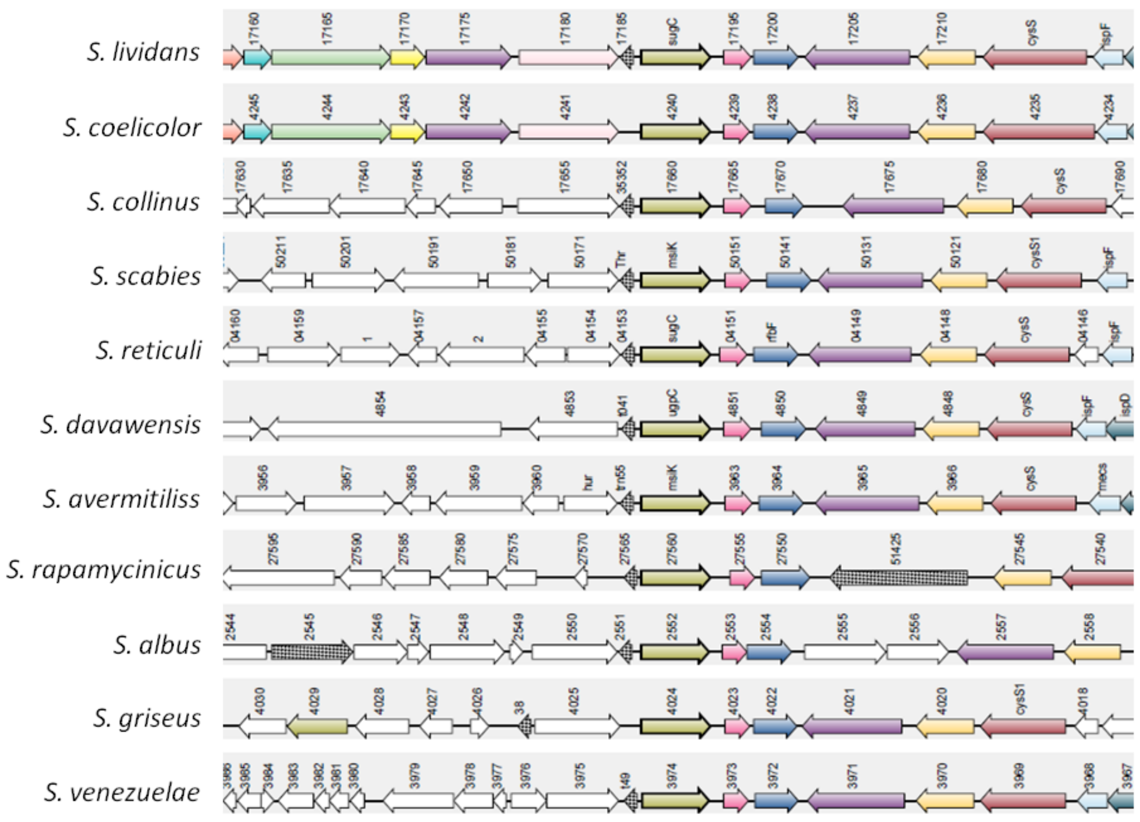

Synteny of the *msiK* locus in a series of *Streptomyces* species. The amino acid sequence of MsiK of *S. lividans* was used as query. The Absynte software identified SCAB_50131 as closest match of MsiK.

Supplementary Figure S3.

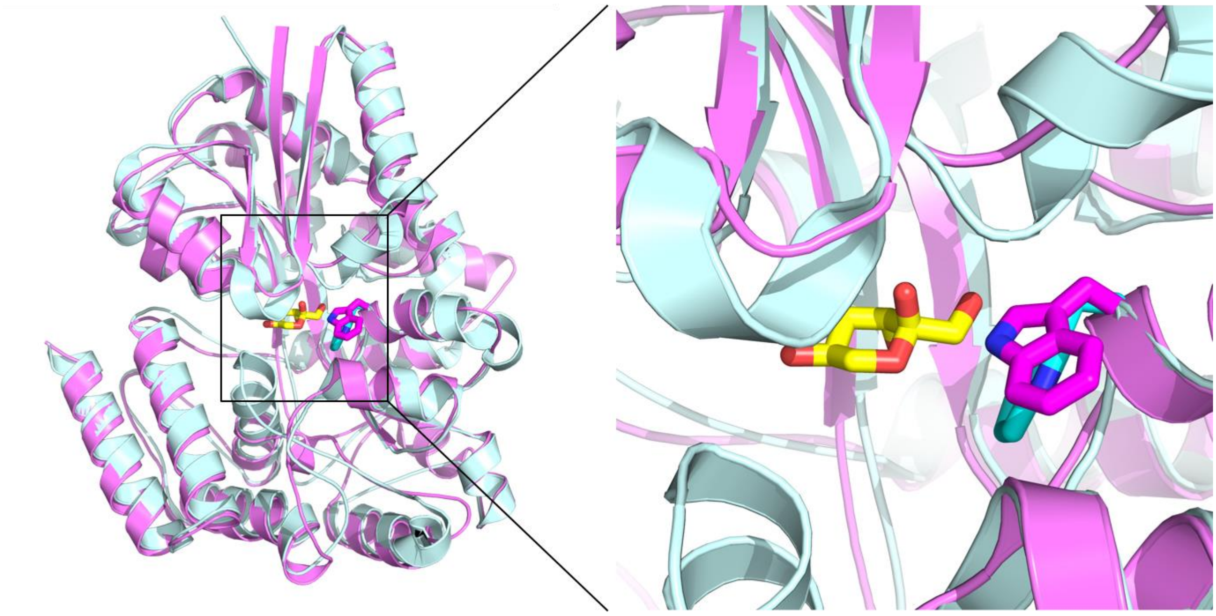

Predicted three-dimensional structure of CebE of *S. scabies*. The X-ray structure of the ABC transporter solute binding protein from *Thermotoga Lettingae* TMO (PDB code: 5CI5, cyan) with an alpha-D-Tagatose molecule (yellow sticks) in its binding pocket is superimposed on the CebE model (magenta) obtained with Yasara. CebE Trp303 and its equivalent in 5CI5 are displayed as sticks.

Supplementary Figure S4.

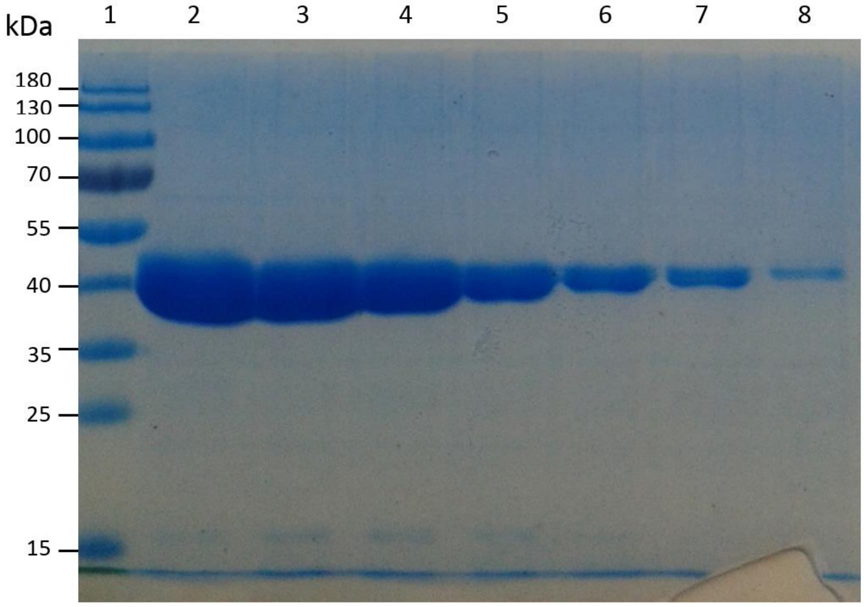

SDS-PAGE showing the level of purity of His6-tagged CebE protein. Lane 1, molecular weight marker (Fermentas). Lane 2 to 8, decreasing amounts of 6His-CebE; the level of purity was estimated to 99%.

Supplementary Figure S5.

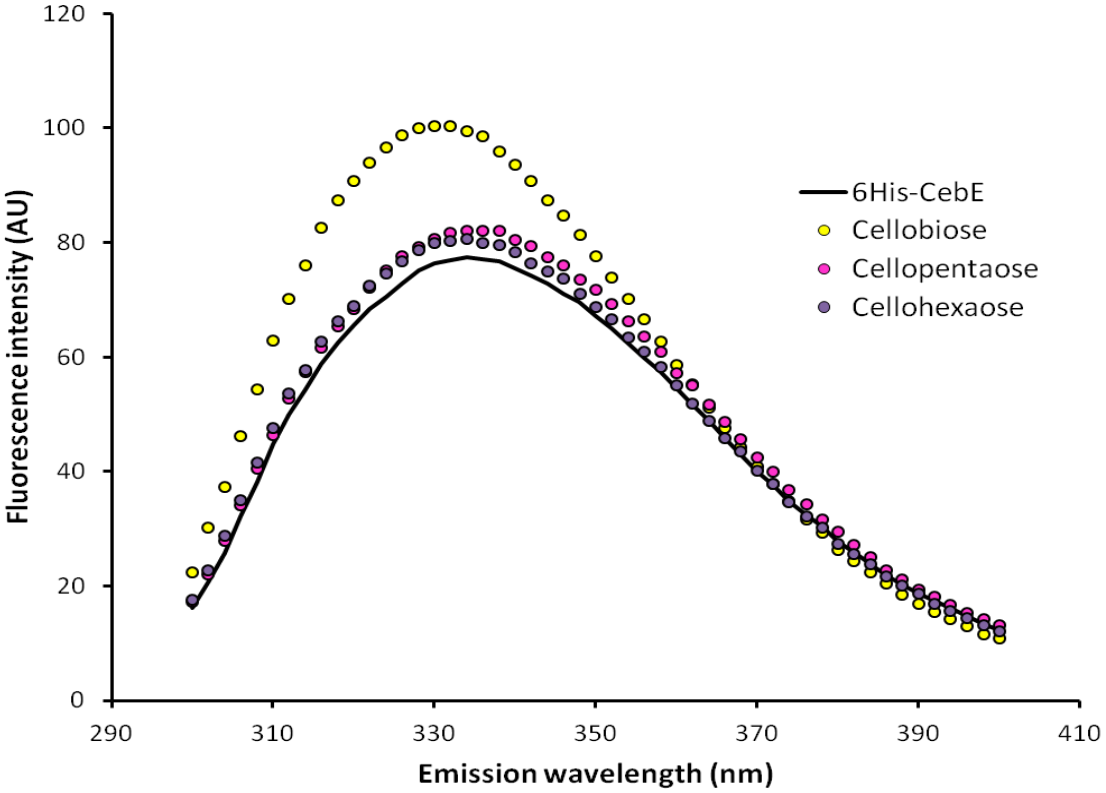

CebE of *S. scabies* does not interact with cellopentaose and cellohexaose. Fluorescence emission spectra of the pure 6His-CebE protein (180 nM) alone (line) or in presence of 30 µM cellobiose (yellow circles), cellopentaose (pink circles) or cellohexaose (violet circles).

Supplementary Figure S6.

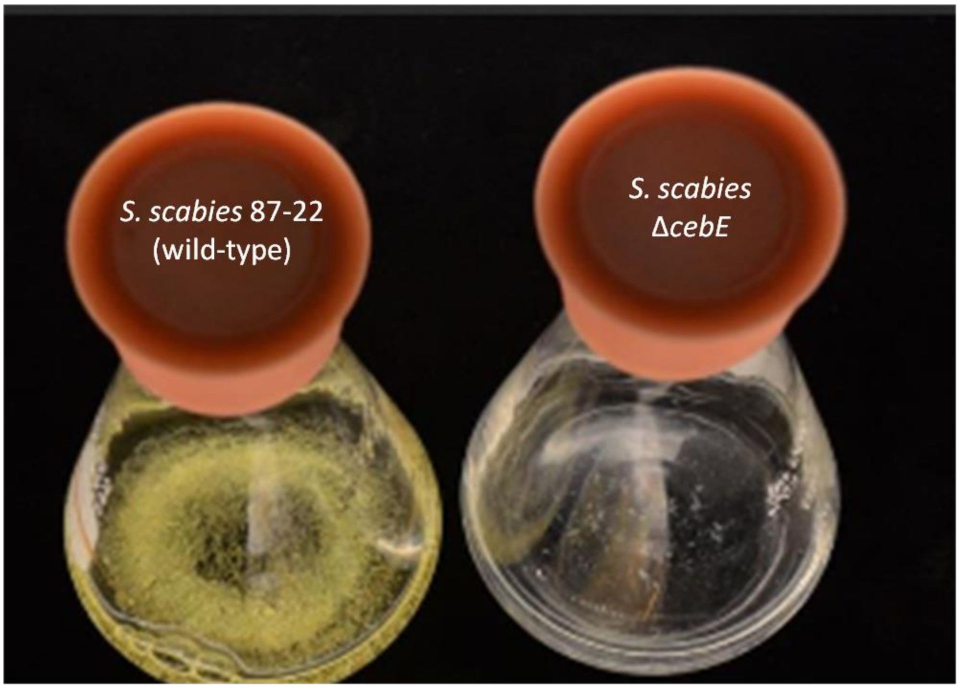

Effect of *cebE* deletion on *S. scabies* growth in liquid TDM supplied with cellobiose as sole carbon source. Note the impaired growth of the Δ*cebR* mutant in liquid medium supplied with cellobiose whereas the mutant was still able to grow on agar TDM + cellobiose plates (Figure 3A).

Supplementary Figure 7

CebE

Two-sided t-test with equal variance

	ESDYLPWK	SAFDLTAK	SGNWGGSFLSVPK
WT vs WT + $(Glc)_2$	0,009958756	0,003262106	0,002972326
WT vs Δ*cebR*	0,000131794	0,001183695	0,000591562
WT + $(Glc)_2$ vs Δ*cebR* + $(Glc)_2$	0,659215339	0,497253423	0,256759088
Δ*cebR* vs Δ*cebR* + $(Glc)_2$	0,276868802	0,044630554	0,369305413
WT vs Δ*cebR* + $(Glc)_2$	0,00940403	0,000839504	0,008705521

Significant variation

Unsignificant variation

MsiK

Two-sided t-test with equal variance

	ILDLTEYLDR	TQIASLQR	FGNSVVPVNR
WT vs WT + $(Glc)_2$	0,834787999	0,246641608	0,047774836
WT vs Δ*cebR*	0,003155968	0,004186295	0,00507292
WT + $(Glc)_2$ vs Δ*cebR* + $(Glc)_2$	0,055086199	0,038580848	0,096400857
Δ*cebR* vs Δ*cebR* + $(Glc)_2$	0,139238958	0,117778972	0,920927647
WT vs Δ*cebR* + $(Glc)_2$	0,045281001	0,013320188	0,050678066

Targeted proteomics overexpression statistical significance. Two-sided t-tests to groups assumed of equal variances. Statistical significance was assigned to $p<0.05$.

Supplementary Method

Detailed protocol of the targeted proteomic analysis.

LC-MS^E analysis

The protein lysate was mixed with 100% (w/v) TCA solution (Sigma-Aldrich) (4:1). The extract was then centrifuged at 16,000 x g for 30 min at 4°C. The supernatant was discarded and the pellet was washed twice with ice-cold acetone (Sigma-Aldrich). After each washing step the pellet was recovered by centrifugation at 16,000 x g for 30 min at 4°C. The resulting

pellet was solubilized in 50 mM ammonium bicarbonate (Sigma-Aldrich) containing 2 M urea (GE Healthcare). The protein concentration was assessed by Bradford analysis using the Pierce™ Coomassie Protein Assay Kit. Protein solutions (10 µg) were subsequently reduced, alkylated and digested with trypsin (Promega) overnight (1:50 w/w). Digested samples were dried and dissolved in 50 µl 20mM ammonium formate. This procedure was performed for three biological replicates.

Peptide mixtures (0.2 µg/µl in 20 mM ammonium formate, pH 10) were separated on a NanoAcquity UPLC® system (Waters Corporation) in 2D mode. For the first dimension (high pH) the sample (1 µg) was loaded onto an XbridgeTM BEH130 C18 column (300 µm × 50 mm, 5 µm; Waters) at 3% solvent B1 (A1 & B1: 20 mM ammonium formate in water and ACN respectively, pH 10) at 2 µl/min. Peptides were eluted from the first dimension column in 5 fractions (11.1%, 14.5%, 17.4%, 20.8%, and 45.0% of solvent B1), and fractions were trapped on a Symmetry® C18 trapping column (180 µm × 20 mm, 5 µm; Waters). For the second dimension (low pH) each fraction was separated on a HSS T3 C18 analytical column (75 µm × 250 mm, 1.8 µm; Waters) at 40°C at 250 nl/min by increasing the acetonitrile concentration from 5 to 50% B2 (A2 & B2: 0.1% formic acid in water and ACN respectively, pH 2) over 60 min. The outlet of the column was directly connected to a PicoTip™ Emitter (New Objective) mounted on a Nanolockspray source of a SYNAPT™ G1 HDMS mass spectrometer (Waters). The time- of-flight (TOF) analyzer was externally calibrated with MS/MS fragments of human [glu1]-fibrinopeptide B (Glu-fib) from m/z 72 to 1285, and the data were corrected post-acquisition using the monoisotopic mass of the doubly charged precursor of Glu-fib (m/z 785.8426) (lock mass correction). Accurate mass data were collected in a data independent positive mode of acquisition (MS^E) by alternating between low (5 V) and high (ranging from 15 to 35 V) energy scan functions (Geromanos et al., 2009). The selected m/z range was 125–2000 Da. The capillary voltage was set to 3.0 kV, the

sampling cone voltage was 26 V and the extraction cone voltage 2.65 V. The source temperature was set at 65°C.

The acquired spectra (Waters.raw) were loaded to the Progenesis software (version 4.1, Nonlinear) for label free quantification. Peaks were then modeled in non-noisy areas to record their peak m/z value, intensity, area under the curve (AUC) and m/z width. After selecting a quality control sample (mixture of all samples) as a reference, the retention times of all other samples within the experiment are aligned to maximal overlay of the 2D feature maps. After alignment and feature exclusion, samples were divided into the appropriate condition. The identification was done by ProteinLynx Global SERVER v2.5 (PLGS, Waters Corporation). The precursor and fragment ion tolerance were determined automatically. The default protein identification criteria used included a maximal protein mass of 250,000 Da, a detection of minimal of three fragment ions per peptide, minimal seven fragment ions per protein and minimal two peptides per protein. Carbamidomethyl-cysteine (fixed) and methionine oxidation (variable) were selected as modifications. Maximally one missed cleavage and a false positive rate of 4% was allowed. After normalization of the raw abundances of all features and statistical analysis the different fractions were combined. For quantification, all unique peptides of an identified protein were included and the total cumulative abundance was calculated by summing the abundances of all peptides allocated to the respective protein. ANOVA values of $p < 0.05$, regulation of 1.5-fold or 0.66-fold, power > 0.8 and q-values < 0.05 were regarded as significant for all further results.

Liquid Chromatography-Multiple Reaction Monitoring (LC-MRM) Analysis

The protein sample preparation and quantitation were similar to those for the $LCMS^E$. The protein lysatedescribed above was mixed with 100% (w/v) TCA solution (Sigma-Aldrich) (4:1). Protein solutions (50 µg) were spiked with 200 ng BSA (Protea Biosciences Group), reduced, alkylated and digested overnight with 0.5 µg trypsin (Promega). Digested samples

were dried and dissolved in 500 µl 0.1% formic acid (Biosolve) in water for LC-MRM analysis (5 µl injection). This procedure was performed for three biological replicates.

The samples were subjected to RPLC on a U3000-RSLC system (Thermo). The samples were loaded onto a Thermo Acclaim® PepMap100 C18 pre-concentration column (2 cm x 100 µm, 5 µm, 100 Å) at a flow rate of 5 µl/min and flushed for 5 min with 0.1% HCOOH/2% ACN. The sample was then separated on a Thermo Acclaim® PepMap100 C18 analytical column (25 cm x 75 µm, 3 µm, 100 Å) at a flow rate of 300 nl/min during a 30 min linear gradient, going from 2 to 40% solvent B, with solvent A (0.1% HCOOH in water) and solvent B (0.1% HCOOH in ACN). The eluting peptides were sprayed directly in a 4000 QTRAP mass spectrometrer (AB Sciex) with a NanoSpray II ESI source (AB Sciex) using a PicoTip™ Emitter (New Objective). The ion spray voltage, curtain gas and nebulizing gas were respectively set at 3.5 kV, 10 and 5 (arbitrary units). The interface heater temperature was fixed at 60°C.

Targeted peptides (two for each protein) were measured in multiple reaction monitoring (MRM) acquisition mode with a dwell time of 100 ms and maximum total cycle time of 3 s. The double charged peptide was selected as precursor (Q1), fragmented (Q2) and for each precursor three fragment ions (y-ions) were selected (Q3). The MRM data were imported in Skyline v3.1 and subjected to a Savitsky-Golay Smoothing transformation. The total area under the curve (AUC) of each targeted peptide was exported and normalized to the spiked BSA standard. A student's t-test (two-tailed, homoscedastic) was performed to evaluate the significance of the differential protein abundance levels between the different conditions.
